# Anti-inflammatory HDL effects are impaired in atrial fibrillation

**DOI:** 10.1007/s00380-021-01908-w

**Published:** 2021-08-30

**Authors:** Erik Holzwirth, Tina Fischer-Schaepmann, Danilo Obradovic, Mirjam von Lucadou, Edzard Schwedhelm, Günter Daum, Gerhard Hindricks, Gunther Marsche, Markus Trieb, Holger Thiele, Jelena Kornej, Petra Büttner

**Affiliations:** 1 Department of Internal Medicine/Cardiology, Heart Center Leipzig at University of Leipzig, Strümpellstr. 39, 04289 Leipzig, Germany; 2 Institute of Clinical Pharmacology and Toxicology, University Medical Centre Hamburg-Eppendorf, Hamburg, Germany; 3 DZHK (German Centre for Cardiovascular Research), Partner Site Hamburg/Kiel/Lübeck, Hamburg, Germany; 4 Department of Vascular Medicine, University Medical Centre Hamburg-Eppendorf, Hamburg, Germany; 5 Department of Electrophysiology, Heart Center Leipzig at University Leipzig, Leipzig, Germany; 6 Division of Pharmacology, Otto Loewi Research Center, Medical University of Graz, Graz, Austria; 7 School of Medicine-Cardiovascular Medicine, Boston University, Boston, MA USA

**Keywords:** Atrial fibrillation, HDL function, Inflammation, Sphingosine 1 phosphate, Renin angiotensin aldosterone system

## Abstract

High-density lipoprotein (HDL), best known for cholesterol transport, also has anti-inflammatory effects. Previous studies suggest involvement of myeloperoxidase (MPO) in modification of HDL. HDL bound Sphingosine-1-phosphate (S1P) has been implied to be an essential protein regarding beneficial HDL effects. In this study, we analyzed anti-inflammatory HDL properties in patients with atrial fibrillation (AF), a disease involving atrial inflammation, compared to non-AF controls and whether anti-inflammatory properties improve upon catheter ablation. Additionally, association with serum concentrations of MPO and S1P were assessed. We isolated HDL from 25 AF patients, 13 non-AF individuals and 14 AF patients at follow-up (FU) after catheter ablation. S1P was measured in a cohort of 141 AF and 21 FU patients. Following preincubation with HDL from either group, bovine aortic endothelial cells were stimulated using tumor necrosis factor α and expression of pro-inflammatory genes intercellular adhesion molecule 1 (ICAM1), vascular cell adhesion molecule 1 (VCAM1), E-selectin (SELE) and P-selectin (SELP) was assessed using qPCR. Concentrations of circulating protein of these genes as well as MPO and S1P were measured in serum samples. Compared to non-AF individuals HDL from AF patients suppressed gene expression of the pro-inflammatory adhesion molecules ICAM1, VCAM1, SELE and SELP 27%, 18%, 21% and 57% less, respectively (*p* < 0.05 for all except SELE *p* = 0.06). In FU patients, the anti-inflammatory HDL activity was improved (suppression of ICAM1 + 22%, VCAM1 + 10%, SELE + 38% and SELP + 75%, *p* < 0.05 for all except VCAM1 *p* = 0.08). AF patients using angiotensin converting enzyme inhibitors or angiotensin receptor blockers had better anti-inflammatory HDL properties than non-users (gene expression suppression at least 28% more, *p* < 0.05 for all except ICAM1 *p* = 0.051). Circulating protein concentrations were not correlated with in vitro gene-expression, but circulating P-selectin was generally elevated in AF and FU patients compared to non-AF patients. MPO plasma concentration was positively associated with gene-expression of ICAM1, VCAM1 and SELP (*r*^2^ > 0.4, *p* < 0.05). Serum concentrations of S1P were increased in FU patients {1.201 µM [1.077–1.543]} compared to AF patients {0.953 µM [0.807–1.135], *p* < 0.01} but not correlated with ICAM1, VCAM1 and SELP gene expression. We conclude that the anti-inflammatory activity of HDL is impaired in AF patients, which might promote AF progression and AF-associated complications.

**Supplementary Information:**

The online version contains supplementary material available at 10.1007/s00380-021-01908-w.

## Introduction

Atrial fibrillation (AF) is a progressive arrhythmia with a lifetime prevalence of one in three among individuals of European ancestry [1, 2]. Cardiovascular complications, such as heart failure, stroke, systemic emboli [3], increased incidence of dementia [4], remain a substantial health hazard in AF patients.

Previous results from the Framingham heart study showed an inverse correlation of high-density-lipoprotein (HDL) concentrations and cardiovascular mortality [5]. Subsequent attempts, however, to raise HDL-cholesterol levels pharmacologically failed to improve patient outcomes [6, 7]. Based on these observations, current HDL research focuses more on HDL functionality rather than HDL-cholesterol levels alone. Beyond its role in the reverse cholesterol transport, HDL and its major apolipoproteins ApoA-I and ApoM [8], exhibit cardioprotective properties, notably by attenuating inflammatory processes [9, 10]. Furthermore, the anti-inflammatory HDL functionality is also mediated by HDL-associated sphingosine-1-phosphate (S1P) bound to apoM [11].

Recently, we demonstrated that key parameters of HDL functionality including lecithin-cholesterol acyltransferase activity, ApoA-I content and HDL particle numbers, are markedly reduced in AF [12]. Importantly, restoration of sinus rhythm ameliorated HDL dysfunction in AF patients after catheter ablation [12].

Previous studies demonstrated that functional HDL inhibits the tumor necrosis factor α (TNF-α)-induced gene-expression of pro-inflammatory endothelial cell surface molecules such as vascular cell adhesion molecule 1 (VCAM1), intercellular adhesion molecule 1 (ICAM1) and E-selectin (SELE) [9, 10]. P-selectin (SELP) has been implicated in AF complications as its concentration is associated with severity of echocardiographic left atrial spontaneous contrast and the presence of left atrial appendage thrombus [13] and is regulated by TNF-α [14]. However, potential regulatory HDL effects have not been reported yet.

AF incidence appears to be decreased in patients using angiotensin converting enzyme inhibitors (ACE-I) or angiotensin receptor blockers (ARB) [15]. While the underlying mechanisms are not fully understood, neurohumoral antagonism and beneficial effects on structural and electrical remodelling have been proposed as potential mechanisms of action [16]. Importantly, ACE-I/ARB have been shown to be protective regarding HDL functionality [17], suggesting a novel beneficial mechanism in the pathophysiology of AF.

In this study, we compared the anti-inflammatory functionality of HDL derived from AF patients and non-AF controls without overt heart diseases. We further tested whether sinus rhythm restoration or the use of ACE-I/ARB are associated with anti-inflammatory functionality of HDL.

## Methods

### Study population

#### AF cohort

Patients undergoing first AF radiofrequency ablation at the Heart Center Leipzig at the University of Leipzig were recruited between October 2015 and April 2017 as previously described [18]. Exclusion criteria were: age < 18 or > 75 years, pregnancy, acute or systemic inflammatory diseases, valvular AF (valvular heart disease > 2nd degree or surgical valve replacement/repairment), any cancer. Transthoracic and transesophageal echocardiography were performed in all patients prior to intervention.

#### Non-AF cohort (controls)

Non-AF individuals were recruited at the cardiology outpatient clinic at the Heart Center Leipzig at the University of Leipzig beginning in 2018 [12]. Exclusion criteria were as follows: age < 18 years, cardiac disease (coronary artery disease, valvular heart disease > 2nd degree, cardiomyopathy, acute coronary syndrome, unstable angina pectoris, heart failure, any history of myocardial infarction, sudden cardiac death or thrombo-embolic event, supraventricular/ventricular arrhythmia), any cancer, renal impairment (eGFR < 30 ml/min), liver dysfunction, acute or systemic infections, or any known autoimmune disease.

Both studies were approved by the local Ethical Committee (Medical Faculty, University Leipzig, registration number 128/18-ek and 259-15-13072015, respectively) written informed consent was obtained from all members of either study group.

### Catheter ablation procedure and follow-up examinations

Catheter ablation procedure with isolation of pulmonary veins and additional lesions if required was performed as reported previously [18]. Briefly, the electro-anatomical mapping was performed in sinus rhythm. In patients who presented with AF prior to the ablation procedure, the arrhythmia was terminated by electrical cardioversion and the procedure was further performed in sinus rhythm. Endpoint of catheter ablation was isolation of the pulmonary veins with proof of both exit and entrance block.

After 3, 6 and 12 months following the ablation, 4-day Holter ECG recordings were registered, whereas the first 3 months were considered as a blanking period. Additional ECGs and Holter ECG recordings were obtained if patients’ symptoms were suggestive of AF. Recurrences were defined as any atrial arrhythmia lasting > 30 s.

### Determination of sample sizes

Based upon our prior observations, that lecithin-cholesterol acyltransferase activity is significantly reduced in AF patients compared to non-AF individuals (28.11% ± 6.27 vs. 38.13% ± 5.52, *p* < 0.001) [12], we assumed a similar effect size for anti-inflammatory HDL activity. G-Power software was used to calculate group sizes based on an allocation rate of 2 to 1, a power of 0.95 and an α error probability of 0.05. This analysis resulted in suggested group sizes of at least 13 AF patients and 7 non-AF individuals.

### Blood sampling

Patient blood samples were withdrawn before AF catheter ablation from the femoral vein using Sarstedt serum S-Monovettes or EDTA Monovettes after > 8 h fasting period. FU blood was collected 12–18 months following the ablation procedure during a follow-up visit. Blood from non-AF individuals and FU samples were taken from a cubital vein. All blood samples were processed within 1 hour after blood draw. For serum, blood was allowed to clot before centrifugation (1800 g, 10 min, 4 °C). EDTA plasma was centrifuged at 1000 g for 10 min. Serum and plasma aliquots were stored at − 70 °C until further use.

### HDL isolation

HDL isolation was performed as described before with minor modifications [19]. Briefly, 1.5 ml of patient serum was diluted 1:2 in phosphate buffered saline (PBS) before potassium bromide (KBr, pro analysis grade, Carl Roth, Karlsruhe, Germany) was added to obtain a density of 1.24 g/ml. Ultracentrifugation tubes (Ultracrimp, Thermo Fisher, Waltham, USA) were filled with 9 ml KBr solution (*ρ* = 1.063 g/ml) and the sample was cautiously syringed to the bottom of the ultracentrifugation tube using a 21 G needle. Ultracentrifugation was performed at 415.000 g for 6 h at 15 °C.

HDL bands were visible as yellow band after centrifugation and were carefully withdrawn. Desalting columns (PD-10 Desalting Columns, GE Healthcare, Chicago USA) were used to remove KBr residues and ultrafiltration tubes (Vivaspin Turbo 15, Sartorius, Göttingen, Germany) were used to concentrate HDL samples. Isolated HDL was stored at 4 °C and used within 12 h to avoid storage-related changes in HDL composition and functionality [19].

### HDL concentration measurements

A standard curve using bovine serum albumin was prepared and HDL protein concentrations were determined using the UV–Vis method (Infinite 200 Pro, Tecan, Männedorf, Switzerland). HDL concentration was adjusted to 0.25 mg/ml with PBS.

HDL purity was exemplarily confirmed by gel electrophoresis and Coomassie staining using 50 µg of HDL isolate and Western Blot analysis using antibodies against ApoA-I (order number: ab7613, Abcam, Cambridge, England).

### Cell culture

All cell culture experiments were performed in at least triplicates using confluent bovine aortic endothelial cells (BOAEC) (PeloBiotech, Planegg, Germany). We chose BOAEC for technical reasons as these cells provided superior reproducibility compared to human aortic endothelial cells [20, 21]. BOAECs were cultured using medium provided by the distributor. Cells were passaged before reaching confluency and experiments were performed following their 6th passage in 24-well plates. 50 µg/ml HDL was carefully mixed with 36 °C cell culture medium and then added to BOAECs for a 12-h incubation. Then 10 ng/ml recombinant tumor necrosis factor-α (TNF-α, Waltham, MA, USA) was added for another 12 h. Cells were then washed with PBS once and harvested in RLT lysis buffer supplemented with β-mercaptoethanol for RNA isolation as recommended by the manufacturer (RNeasy Mini Kit, Qiagen, Hilden, Germany).

### RNA isolation and reverse transcription

RNA was isolated using the RNeasy Minikit (Qiagen, Hilden, Germany) according to the manufacturer’s instructions. Reverse transcription of 250 ng total RNA was performed using the Omniscript reverse transcriptase and poly-dT primers (Qiagen, Hilden, Germany) according to the manufacturer’s protocol.

### Quantitative real-time PCR

Exon-exon spanning primer sequences were either chosen based on previous publications [22] or designed using Primer3Plus [23]. HPRT: fw-CTGGCTCGAGATGTGATGAA, rv-CAACAGGTCGGCAAAGAACT, ICAM1: fw-GACTTCTTCAGCTCCCCAAG, rv-CCCACATGCTATTTGTCCTG, VCAM1: fw-GAGCTTGGACGTGACCTTCT, rv-TGGGTGGAGAATCATCATCA, SELE: fw-TGTGAAGCTCCGACTGTGTC, rv-GCGTTTCAGAAGCCAGAAGAG, SELP: fw-ACAACCAGGACTGTGTGGAG, rv-GTCCTGGCAGGAGGCTCTAT. Semi-quantitative real-time-PCR was performed using Takyon Mastermix (Eurogentec, Lüttich, Belgium) using primers at a final concentration of 100 nM on a real-time PCR thermocycler (CFX Connect, BioRad, Hercules, USA).

### Analysis of circulating ICAM1, VCAM1, SELE and SELP

Levels of ICAM1, VCAM1, SELE and SELP in EDTA plasma were measured using an antibody-based Luminex multiplex screening assay (R&D Systems/bio-techne, Minneapolis, MN, USA) and a Luminex 200 system (Merck, Darmstadt, Germany). All samples were diluted 1:2 before analysis.

### Myeloperoxidase (MPO) ELISA

Plasma samples were diluted 1:10 and MPO concentrations were determined using a commercially available Elisa kit (RHK324, BioVendor, Brno, Czech Republic) according to the manufacturers’ recommendations.

### S1P measurements

S1P was measured in serum of 141 AF patients whereas 21 had FU samples and data accessible. S1P was determined by LC-MS/MS as previously described [24]. In brief, 20 µL of serum sample were incubated with 20 µL of internal standard 1 µM [16,17,18-^2^H_7_]-S1P (S1P-d_7_, Avanti Polar Lipids, Alabaster, AL, USA) and subsequently proteins were precipitated by addition of 350 µL of acetonitrile/water, 80/20 (vol/vol). The supernatant was subjected to reverse-phase chromatography on a Zorbax SB-C8 (2.1 × 50 mm; Agilent Technologies, Santa Clara, CA, USA). S1P was eluted with 0.35 ml/min as a binary gradient for 6 min (methanol/acetonitrile/0.1% formic acid: 2.5/2.5/95–30/30/40, vol/vol/vol) and quantified by tandem mass spectrometry (Varian L1200 MS/MS, Agilent Technologies, Waldbronn, Germany).

### Statistical analyses

PCR replicates with quantification cycle (Cq) standard deviations > 0.25 or with housekeeper Cq above 30 (typical range was 25.3–28.5, mean Cq 26.6 ± 0.74) were excluded from further analysis. Gene-expression was normalized to HPRT gene-expression. Calculations were performed using the CFX Maestro software (BioRad, Hercules, USA) utilizing the ΔΔCq method. Relative gene-expression in % was calculated using gene-expression after TNF-α stimulation as a baseline set to 100%. Statistical analyses were performed using SPSS V24 software (IBM). Continuous variables were tested for normal distribution using the Kolmogorov–Smirnov test. Student’s *t* test or Mann–Whitney *U* test were used to determine group differences for continuous variables for parametric and non-parametric data sets, respectively. Binary data were compared using Chi-Square testing. Graphs and figures were created with GraphPad Prism 8 (GraphPad Software Inc). Power calculations were carried out using GPower (University of Düsseldorf, Germany). Normally distributed data are presented as mean ± standard deviation, non-normally distributed data as median and interquartile ranges. A *p* value < 0.05 was considered statistically significant.

## Results

### Cohorts

In total, 141 AF patients and 13 non-AF individuals were recruited (Table 1). For cell culture experiments and measurements of circulating ICAM1, VCAM1, SELE, SELP and MPO protein a subgroup of 25 AF patients before catheter ablation (median age 62 years, 44% women), 13 non-AF patients (median age 62 years, 54% women) and 14 non-matched AF patients after catheter ablation during FU was analysed whereas sufficient sample accessibility for HDL-isolation was a selection criterion. There were 14 AF patients with available samples during follow-up (median age 65 years, 57% women). Seven (50%) of these patients had an AF recurrence (Table 2). S1P measurements were performed in 141 AF patients and 21 FU patients. Among those 21 FU patients 6 (28.6%) presented with recurrent AF. We did not observe any statistical differences between AF patients and non-AF patients regarding age, sex, BMI, prevalence of hypertension and diabetes or usage of ACE-I/ARB (Table 1).

**Table 1:** Clinical characteristics of the study groups

Testing of HDL functionality in vitro and determination of circulating proteins			
*n*	Non-AF 13	AF 25	p-Values
Age (years)	62 [61–63]	62 [58–69]	0.632^a^
Female (%)	53.8	44.0	0.564^b^
aHT (%)	84.6	88.0	0.770^b^
Diabetes (%)	30.8	16.0	0.289^b^
BMI (kg/m^2^)	31 [26–34]	28 [27–34]	0.988^c^
LA size (mm)		44 [39–48]	n.a
LV-EF (%)		56 [48–61]	n.a
ACE-I/ARB (%)	76.9	64.0	0.416^b^
Persistent AF (%)	n.a.	60.0	n.a.

S1P measurements			
*n*	AF 141	FU 21	
Age (years)	65 [56–72]	65 [55–67]	0.403^a^
Female (%)	43.3	61.9	0.110^b^
aHT (%)	78.0	71.4	0.502^b^
Diabetes	18.4	23.8	0.559^b^
BMI (kg/m^2^)	29 [26–33]	30 [26–33]	0.516^a^
ACE-I/ARB (%)	68.8	61.9	0.529^b^
Persistent AF (%)	53.2	57.1	0.735^b^

*AF* atrial fibrillation, *FU* follow-up, *aHT* arterial hypertension, *BMI* body mass index, *ACE-I* angiotensin converting enzyme inhibitor, *ARB* angiotensin receptor blocker, *LV-EF* left ventricular ejection fraction, *n.a.* not applicable

**Table 2:** Gene-expression of ICAM1, VCAM1, SELE and SELP in endothelial cells stimulated with TNF-α and incubated with HDL from patients with restored sinus rhythm or with AF recurrence at FU

Gene of interest	AF recurrence *n* = 7	Sinus rhythm restoration *n* = 7	*p* value
ICAM1 (%)	41.2 [25.6–57.6]	28.9 [20.9–38.5]	0.180
VCAM1 (%)	20.3 [15.8–23.3]	16.5 [13.7–20.1]	0.142
SELE (%)	20.1 [16.5–34.2]	15.9 [13.6–36.5]	0.655
SELP (%)	13.1 [7.7–19.9]	7.3 [6.2–14.8]	0.482

TNF-α stimulation without the addition of HDL was set 100%, *p* values were calculated using a paired Mann–Whitney *U* test

### Anti-inflammatory properties of HDL

Stimulation of BOAECs with TNF-α resulted in significantly increased gene-expression levels of all genes of interest compared to non-treated cells (Supplementary Fig. 1).

**Fig. 1:**
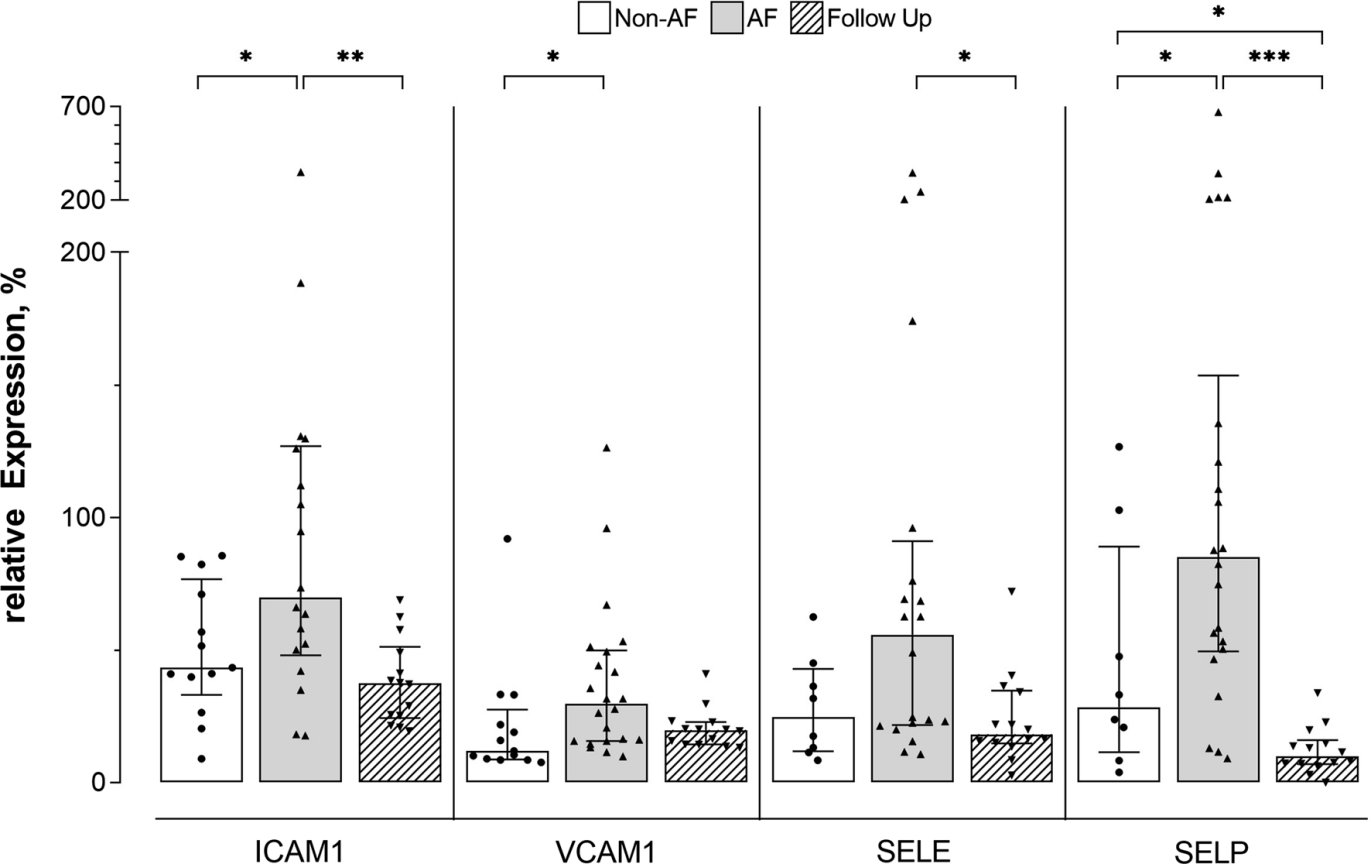
Gene-expression (median [IQR]) of ICAM1, VCAM1, SELE and SELP in endothelial cells stimulated with TNF-α and incubated with HDL from individuals without atrial fibrillation (non-AF), AF patients before catheter ablation (AF) and AF patients after catheter ablation (Follow Up), TNF-α stimulation without the addition of HDL was set 100%, *p < 0.05, **p < 0.01, ***p < 0.001. Statistical analysis was performed using a paired Mann–Whitney *U* test

The addition of HDL isolated from either study group to BOAECs prior to stimulation with TNF-α resulted in significantly lower median gene-expression increase (*p* < 0.05) of all four tested genes in all experimental groups. HDL isolated from non-AF individuals or patients at FU had a significantly stronger effect compared to HDL from AF patients (Fig. 1 and Supplementary Table 1). HDL isolated from some AF patients before ablation further enhanced the stimulatory effect of TNF-α for all target genes. This pro-inflammatory activity of HDL was observed only once in the non-AF group for SELP and was not observed in the FU group.

Compared to HDL from non-AF individuals, the gene-expression of ICAM1 (43.4%, 39.9–71.1, *n* = 13 vs. 69.9%, 50.1–126.0, *n* = 18, *p* = 0.045), VCAM1 (12.0%, 9.1–21.9, *n* = 13 vs. 29.7%, 15.8–49.4, *n* = 22, *p* = 0.017), SELE (24.7%, 12.4–40.7, *n* = 8 vs. 55.8%, 22.0–86.1, *n* = 20, *p* = 0.060) and SELP (28.5%, 14.7–75.2, *n* = 8 vs. 85.1%, 50.5–135.6, *n* = 22, *p* = 0.040) was higher in cells incubated with HDL from AF patients before catheter ablation (Fig. 1).

Compared to HDL from AF patients before ablation, HDL from AF patients at FU significantly decreased the gene-expression of ICAM1 (69.9%, 50.1–126.0, *n* = 18 vs. 37.5%, 25.3–49.1, *n* = 14, *p* = 0.004), SELE (55.8%, 22.0–86.1, *n* = 20 vs. 18.3%, 15.2–34.2, *n* = 14, *p* = 0.012) and SELP (85.1%, 50.5–135.6, *n* = 22 vs. 10.0%, 7.2–14.8, *n* = 14, *p* < 0.001). By trend VCAM1 (29.7%, 15.8–49.4, *n* = 22 vs. 19.8%, 14.5–22.8, *n* = 14, *p* = 0.080) was also decreased in FU patients (Fig. 1 and Supplementary Table 1). This observation was independent of rhythm outcomes in these patients. However, numerically a more pronounced decrease of gene-expression was observed in patients with sinus rhythm restoration (Table 2).

The gene-expression values of all investigated genes correlated significantly with each other in all samples (*p* < 0.0001, *r* > 0.6, Supplementary Table 2).

### Use of ACE-I or ARB

We found that HDL from AF patients using ACE-I/ARB had significantly different effects on gene-expression in TNF-α stimulated BOAECs compared to those patients not using them. In vitro gene-expression of VCAM1, SELE and SELP was significantly lower (*p* < 0.05), while ICAM1 tended (*p* = 0.051) to be lower in AF patients using ACE-I/ARB (Table 3).

**Table 3:** Gene-expression of ICAM1, VCAM1, SELE and SELP in endothelial cells stimulated with TNF-α and incubated with HDL from AF patients using or not using ACE-I/ARB, TNF-α stimulation without the addition of HDL was set 100%, *p* values were calculated using a paired Mann–Whitney *U* test

Gene of interest	ACE-I/ARB	No ACE-I/ARB	*p* value
ICAM1 (%)	60.9 [35.0–94.8] *n* = 10	120.9 [61.8–159.6] *n* = 8	0.051
VCAM1 (%)	18.7 [15.7–31.7] *n* = 14	46.8 [36.7–81.5] *n* = 8	0.020
SELE (%)	23.4 [17.8–68.9] *n* = 12	118.4 [55.8–226.0] *n* = 8	0.031
SELP (%)	67.9 [32.6–100.59] *n* = 14	167.9 [66.6–280.2] *n* = 8	0.024

### Determination of levels of circulating proteins

**Fig. 2:**
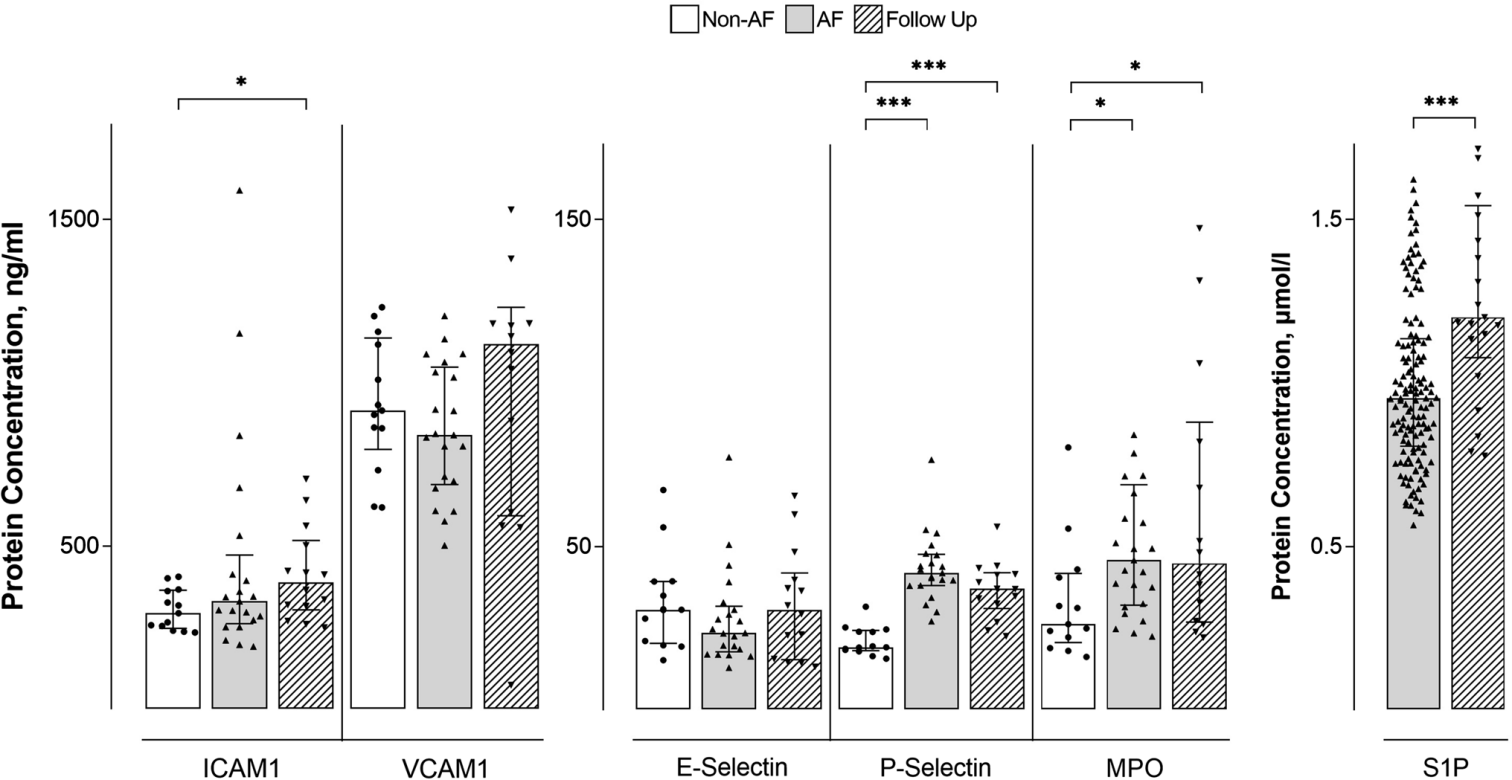
Protein concentrations {median [IQR]} of ICAM1, VCAM1, SELE, SELP, MPO and S1P in individuals without atrial fibrillation (non-AF), AF patients before catheter ablation (AF) and AF patients after catheter ablation (Follow Up). *p < 0.05, ***p < 0.001. Statistical analysis was performed using a paired Mann–Whitney *U* test

Plasma concentrations of ICAM1, VCAM1, SELE and SELP were determined in 13 non-AF individuals, 21 AF patients before catheter ablation, and 14 AF patients after catheter ablation during FU. MPO concentration was measured in 13 non-AF individuals, 25 AF patients before catheter ablation and 14 AF patients after catheter ablation during FU. Both MPO and SELP concentrations were significantly lower in non-AF individuals compared to AF patients before catheter ablation (26 ng/ml, 22–40 vs. 48 ng/ml, 32–72, *p* = 0.010 and 19 ng/ml, 18–24 vs. 42 ng/ml, 38–47, *p* = 0.001) and after catheter ablation during FU (26 ng/ml, 22–40 vs. 45 ng/ml, 27–82, *p* = 0.037 and 19 ng/ml, 18–24 vs. 37 ng/ml, 32–42, *p* = 0.001). Circulating ICAM1 protein levels were significantly higher in AF patients after catheter ablation during FU compared to non-AF individuals (388 ng/ml, 315–501 vs. 294 ng/ml, 254–362, *p* = 0.020, Fig. 2 and Supplementary Table 3).

MPO plasma protein concentrations were positively correlated with in vitro relative gene-expressions of ICAM1, VCAM1 and SELP (*p* < 0.05, *r* > 0.4). MPO concentrations were not different between patients treated or not treated with ACE-I/ARB.

S1P concentration was determined in serum of 141 AF patients before catheter ablation and in 21 of them at FU. S1P levels in AF patients were 0.953 µM (0.807–1.135) at baseline and 1.201 µM (1.077–1.543) at FU (*p* < 0.01, Fig. 2). S1P at FU was comparable in patients in sinus rhythm and with AF recurrence (1.201 µM (0.916–1.573) vs. 1.247 µM (1.176–1.512), *p* = 0.733).

## Discussion

The main finding of our analysis is that anti-inflammatory properties of HDL are impaired in patients with AF compared to non-AF individuals. This finding is in line with the recent observation of decreased HDL-cholesterol efflux capacity, HDL-particle number, apoA-I levels, and lecithin-cholesterol acyltransferase activity in the same patient cohort [12]. Strikingly, HDL functionality was ameliorated in AF patients after catheter ablation during follow-up irrespective of rhythm outcome. While decreased functionality of patient-derived HDL is reflected by a decreased ability of HDL to suppress inflammatory gene-expression induced by TNF-α in endothelial cells in vitro, circulating plasma levels of these molecules remain unchanged. Interestingly, we observed that HDL from AF patients treated with ACE-I/ARB exhibited higher anti-inflammatory HDL functionality than HDL from untreated patients. At last, baseline serum S1P levels were lower in AF patients before catheter ablation compared to non-AF controls and increased at follow-up indicating a pathomechanistic role for S1P in AF.

### Anti-inflammatory function of HDL in atrial fibrillation

During inflammation, expression of pro-inflammatory cell surface molecules such as SELE, ICAM1 and VCAM1 in endothelial cells is upregulated [25]. These molecules are involved in the recruitment of immune cells during the initiation and maintenance of inflammatory processes. As endothelial dysfunction and the associated chronic inflammation are a well described phenomena in AF [26] we chose endothelial cells over other cardiac tissues as a model of the atria for our experiments. Inflammatory hallmarks of AF include increased macrophage infiltration and cytokine deposition into atrial tissue [27] and elevated levels of circulating interleukin-6 [28], TNF-α [29], and MPO [30, 31]. Aside from stimulating the expression of pro-inflammatory cytokines, TNF-α has been found to be a driver of chronic electro-anatomical remodelling [32] as well as short-term alterations in ion currents [33]. Interestingly, other diseases featuring chronic sterile inflammation and pathophysiological involvement of TNF-α including rheumatoid arthritis or psoriasis are associated with increased AF incidences [32]. SELP is stored together with von Willebrand factor (vWF) in endothelial granules, which are secreted upon thrombogenic stimuli and possibly contribute to AF complications [13].

To investigate the anti-inflammatory properties of HDL, we used TNF-α stimulated endothelial cells and measured the potential of HDL to suppress the gene-expression of adhesion molecules. Prior to the pro-inflammatory TNF-α stimulus, cells were pre-incubated with HDL mimicking the constant exposure to bloodstream HDL within the atria in-vivo. We observed a strong correlation of gene-expression between the four genes among all samples indicating their common regulation by TNF-α that includes the activation of NF-κB [34] (Supplementary Fig. 2).

We found that HDL of AF patients was less capable to reduce pro-inflammatory responses to TNF-α stimulation compared to non-AF individuals.

### Possible mechanisms of HDL impairment

It has been postulated that HDL dysfunction may be due to post-translational modifications induced by oxidative stress as a result of MPO activity [35]. Previously, HDL of AF patients was found to feature an elevated extent of oxidation compared to controls [36, 37]. Post-translational modification of HDL by MPO through carbamylation [﻿^38^] or site specific oxidation [35] was shown to render HDL dysfunctional resulting in the loss of its anti-inflammatory activities. Accordingly, we found higher MPO protein concentrations to be correlated with higher pro-inflammatory gene-expression. However, we observed no significant differences in MPO levels between AF patients and FU patients although the anti-inflammatory HDL effects in FU patients had improved. This is in line with results from our previous study where we observed that elevated MPO levels in AF patients are independent from the AF progression phenotype and also the rhythm outcome following catheter ablation [30]. Therefore, we hypothesized that MPO might be involved in the initiation of fibrotic processes but is not directly associated with the extent of fibrotic remodelling in AF [30]. Likewise, MPO may contribute to the observed HDL dysfunction in AF but also other yet unrecognized factors that were not measured in our study may be of importance. Importantly, other common AF comorbidities featuring a state of chronic inflammation including diabetes [﻿^39^], obesity [﻿^40^] or coronary artery disease [﻿^41^] have been linked to alterations in HDL profile and functionality as well.

We found S1P concentrations in AF patients to be within the previously published reference interval in the SHIP-Trend population-based cohort of Eastern Germany, i.e., 0.534–1.242 μmol/l [24]. However, compared to non-AF individuals, AF patients exhibited lower serum-S1P levels. As HDL is a major carrier for circulating S1P it remains to be shown whether lower S1P levels explain the decreased anti-inflammatory HDL properties in AF patients [42]. Interestingly, HDL associated vasodilatation has recently been attributed to its S1P content [11].

We found that in some AF patients HDL not only lost its anti-inflammatory activities but rather increased the expression of the investigated pro-inflammatory genes. This is in line with previous studies suggesting that HDL might exert pro-inflammatory properties as it is capable of incorporating pro-inflammatory proteins like serum amyloid A1 during acute phase reactions [﻿^43^], in acute coronary syndrome and coronary artery disease [41], chronic kidney disease [44], or rheumatoid arthritis [45].

### Catheter ablation procedure for sinus rhythm restoration in AF patients and its effect on HDL functionality

In the present study, we observed that the anti-inflammatory functionality of HDL was improved after catheter ablation. Interestingly, this effect was independent of rhythm outcome during FU examination, an observation that we also made for S1P levels. A contributing factor may be lifestyle changes in FU patients including smoking cessation, blood sugar control, weight loss and exercise that all were found to be beneficial regarding HDL functionality [﻿^46^, ^47^]. However, the relatively small FU sample size did not fulfil the requirements concerning statistical power to analyze the effect of sinus rhythm restoration on anti-inflammatory activity of HDL and should be interpreted with caution.

### Surrogates of HDL functionality

Although anti-inflammatory properties of HDL may represent a powerful marker in AF, its analysis involves a complex in vitro methodology and is thus not feasible in the clinical routine. Here, we addressed the question whether plasma concentrations of fragments of adhesion proteins in AF patients correlate with the anti-inflammatory activities of HDL and might therefore serve as surrogate parameters, but found no correlation of ICAM1, VCAM1, SELE or SELP with anti-inflammatory HDL functionality. SELP concentrations were elevated in AF patients compared to non-AF individuals while levels of ICAM1, VCAM1 and SELE were similar in both groups. We attribute this observation to SELP being a secreted protein, whereas ICAM1, VCAM1 and SELE are membrane-bound proteins fragments which are released into the circulation following proteolytic cleavage. Even severe tissue damage resulting in endothelial activation, as observed during coronary artery bypass surgery, was accompanied by numerically small rise of circulating soluble SELE concentrations only [﻿^48^].

### The use of angiotensin-converting enzyme inhibitor and angiotensin receptor blocker is associated with improvements of HDL functionality

Interestingly, we found that HDL of patients using ACE-I/ARB, had a higher anti-inflammatory functionality compared to HDL from patients not using these drugs. This may indicate a beneficial mechanism, at least in part, that underpins the observed reduced incidence of AF in patients using this medication. Accordingly, we have previously found an inverse association between ACE-I/ARB use and MPO levels in AF [30]. However, this effect was not observable in our current analysis, which could be explained by a smaller sample size (116 patients in our prior study). Importantly, the use of ACE-I/ARB was recently found to ameliorate dialysis-associated HDL dysfunction regarding cholesterol-efflux capacity and the capability to suppress reactive oxygen species production in LPS-activated macrophages [17]. There was no difference in S1P concentrations between patients using or not using ACE-I/ARB in either AF patients before catheter ablation or AF patients at follow up after catheter ablation.

## Limitations

Our data are based on a small sample size, which substantially limits the detectability of small effects. Samples prior to catheter ablation and after catheter ablation at FU were not from the same patients due to limited sample availability. Especially the small number of FU samples limited the analysis of associations between ablation outcome and HDL functionality. Further, the impact of medication other than ACEI or ARB on HDL functionality could not be analyzed. S1P measurements were performed in serum samples of patients from the same cohort that was used for in vitro testing of anti-inflammatory HDL function but due to limited sample accessibility only a small fraction of patients (Baseline *n* = 25 and FU *n* = 5) was present in both experiments. Thus, correlation analyses of in vitro gene-expression levels with S1P concentrations were not feasible. Furthermore, the study cohort included individuals of European ancestry, therefore described results might be different in other ethnicities. Finally, larger clinical and observational studies are needed to prove our hypothesis in a longitudinal setting and to reveal a potential role of HDL dysfunction in common AF comorbidities including dementia or thromboembolic events.

## Conclusions

Our results suggest that the anti-inflammatory activity of HDL is impaired because of AF. As inflammation is an AF hallmark and is involved in tissue remodelling, HDL anti-inflammatory dysfunction may be a contributing pathomechanism underpinning the phenomenon called “AF begets AF”.

Further studies are necessary to assess whether restoration of intrinsic HDL functionality or administration of recombinant HDL might provide beneficial effects on the clinical course of AF.

## Supplementary Information

Below is the link to the electronic supplementary material.

Supplementary file1 (DOCX 72 KB)

Supplementary file2 (JPG 105 KB)

## Abbreviations

ACE-I: Angiotensin converting enzyme inhibitor
AF: Atrial fibrillation
ARB: Angiotensin receptor blocker
BMI: Body mass index
BOAEC: Bovine aortic endothelial cells
Cq: Quantification cycle
eGFR: Estimated glomerular filtration rate
FU: Atrial fibrillation patient at follow-up after catheter ablation
HDL: High density lipoprotein
ICAM1: Intercellular adhesion molecule 1
KBr: Potassium bromide
LC-MS/MS: Liquid chromatography-mass spectrometry/mass spectrometry
LV-EF: Left ventricular ejection fraction
MPO: Myeloperoxidase
PBS: Phosphate buffered saline
S1P: Sphingosine-1-phosphate
SELE: E-selectin
SELP: P-selectin
TNF-α: Tumor necrosis factor α
VCAM1: Vascular cell adhesion protein 1
