# Supplementary material for: Anti-inflammatory HDL effects are impaired in atrial fibrillation
Source: Heart Vessels. 2021 Aug 30;37(1):161–71. doi: 10.1007/s00380-021-01908-w (PMC8732851; doi:10.1007/s00380-021-01908-w)
Supplement: Supplementary file 1 — Supplementary file1 (DOCX 72 KB) [file 380_2021_1908_MOESM1_ESM.docx]

**Supplementary data**

**Anti-inflammatory HDL effects are impaired in atrial fibrillation**

Erik Holzwirth, Tina Fischer, Danilo Obradovic, Mirjam von Lucadou, Edzard Schwedhelm, Günter Daum, Gerhard Hindricks, Gunther Marsche, Markus Trieb, Holger Thiele, Jelena Kornej, Petra Büttner

**Supplementary methods**

Normalized gene expression values in relation to HPRT were calculated using the CFX Maestro software (BioRad, Hercules, USA) and the ΔΔCq method using the following formula:

Normalized Expression = (RQ_(Sample(GOI)))/(RQ_(Sample(HPRT)) )

with

RQ_(Sample(GOI)) = E_GOI^((Cq_min-Cq_sample))

where:

RQ = relative quantity

GOI = gene of interest

Cq_min = Cq for the Sample with the lowest average Cq

Cq_sample = Cq for the sample

E = PCR efficiency, calculated as (Efficiency in % * 0.01 + 1)

**Supplementary Figure 1:** Gene expression of ICAM1, VCAM1, SELE and SELP normalized to HPRT in bovine aortic endothelial cells before (baseline) and after stimulation with TNF-α.


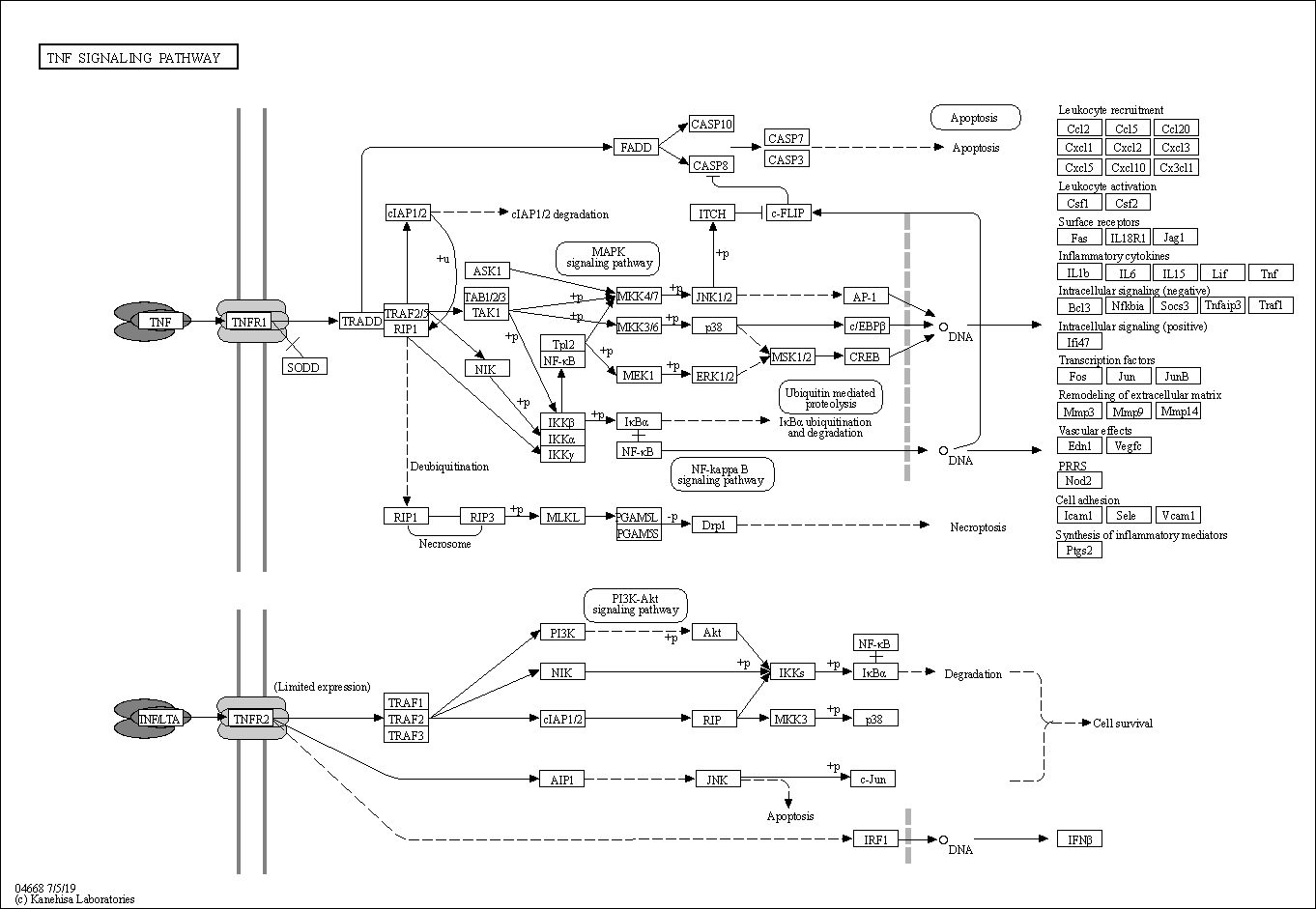


**Supplementary Figure 2:** map04668 – TNF signaling pathway as provided by the Kyoto Encyclopedia of Genes and Genomes (KEGG) (https://www.genome.jp/kegg/kegg2.html) [1], arrows indicate molecular interaction, arrows with +p indicate phosphorylation of the target, ICAM1, SELE and VCAM1 can be found on the right side of the pathway under the heading “Cell adhesion”. Permission to publish the KEGG pathway map was granted by Kanehisa Laboratories.

**Supplementary Table 1:** Expression data for ICAM1, VCAM1, SELE and SELP in endothelial cells stimulated with TNF-α and incubated with HDL from controls without atrial fibrillation (non-AF), AF patients and AF patients 12-18 months after catheter ablation procedure (FU) are shown as median and IQRs. TNF-α stimulation without the addition of HDL was set as 100%. Statistical analysis was performed using a paired Mann-Whitney-U test.

|  |  |  |  | **p - Values** | | |
| --- | --- | --- | --- | --- | --- | --- |
| **Gene of Interest** | **non-AF** | **AF** | **FU** | non-AF  vs AF | non-AF vs FU | AF  vs FU |
| ICAM1, %  n | 43.4  [39.9 – 71.1]  13 | 69.9  [50.1 – 126.0]  18 | 37.5  [25.3 – 49.1]  14 | 0.045* | 0.145* | 0.004* |
| VCAM1, %  n | 12.0  [9.1 – 21.9]  13 | 29.7  [15.8 – 49.4]  22 | 19.8  [14.5 – 22.8]  14 | 0.017* | 0.145* | 0.080* |
| SELE, %  n | 24.7  [12.4 – 40.7]  8 | 55.8  [22.0 – 86.1]  20 | 18.3  [15.2 – 34.2]  14 | 0.060* | 0.838* | 0.012* |
| SELP, %  n | 28.5  [14.7 – 75.2]  8 | 85.1  [50.5 – 135.6]  22 | 10.0  [7.2 – 14.8]  14 | 0.040* | 0.024* | 0.001* |

**Supplementary Table 2**: Correlation of relative gene expressions using Pearson’s r (all p-Values <0.0001)

|  | ICAM1 | VCAM1 | SELE | SELP |
| --- | --- | --- | --- | --- |
| ICAM1 | - | 0.817 | 0.683 | 0.898 |
| VCAM1 | 0.817 | - | 0.727 | 0.788 |
| SELE | 0.683 | 0.727 | - | 0.757 |

**Supplementary Table 3:** Protein concentrations of ICAM1, VCAM1, SELE and SELP measured in plasma from controls without atrial fibrillation (non-AF), patients with AF and AF patients 12-18 months after catheter ablation procedure (FU). Statistical analysis was performed using a paired Mann-Whitney-U test.

|  |  |  |  | **p - Values** | | |
| --- | --- | --- | --- | --- | --- | --- |
| **Protein** | **Non-AF** | **AF** | **FU** | Non-AF  vs AF | Non-AF vs FU | AF  vs FU |
| ICAM1,  ng/ml | 294  [254 – 362] | 330  [273 – 412] | 388  [315 – 501] | 0.330 | 0.020 | 0.381 |
| VCAM1,  ng/ml | 915  [860 – 1116] | 840  [699 – 1031] | 1117  [602 – 1181] | 0.208 | 0.497 | 0.114 |
| SELE,  ng/ml | 31  [21 – 39] | 23  [18 – 31] | 31  [16 – 40] | 0.162 | 0.808 | 0.567 |
| SELP,  ng/ml | 19  [18 – 24] | 42  [38 – 47] | 37  [32 – 42] | 0.001 | 0.001 | 0.055 |
| MPO,  ng/ml | 26  [22 – 40] | 48  [32 – 72] | 45  [27 – 82] | 0.010 | 0.037 | 0.977 |

References

1. Kanehisa, M.; Goto, S. KEGG: kyoto encyclopedia of genes and genomes. *Nucleic Acids Res.* **2000**, *28*, 27–30, doi:10.1093/nar/28.1.27.
