# Supplementary figures and images for: Anti-inflammatory HDL effects are impaired in atrial fibrillation
Source: Heart Vessels. 2021 Aug 30;37(1):161–71. doi: 10.1007/s00380-021-01908-w (PMC8732851; doi:10.1007/s00380-021-01908-w)

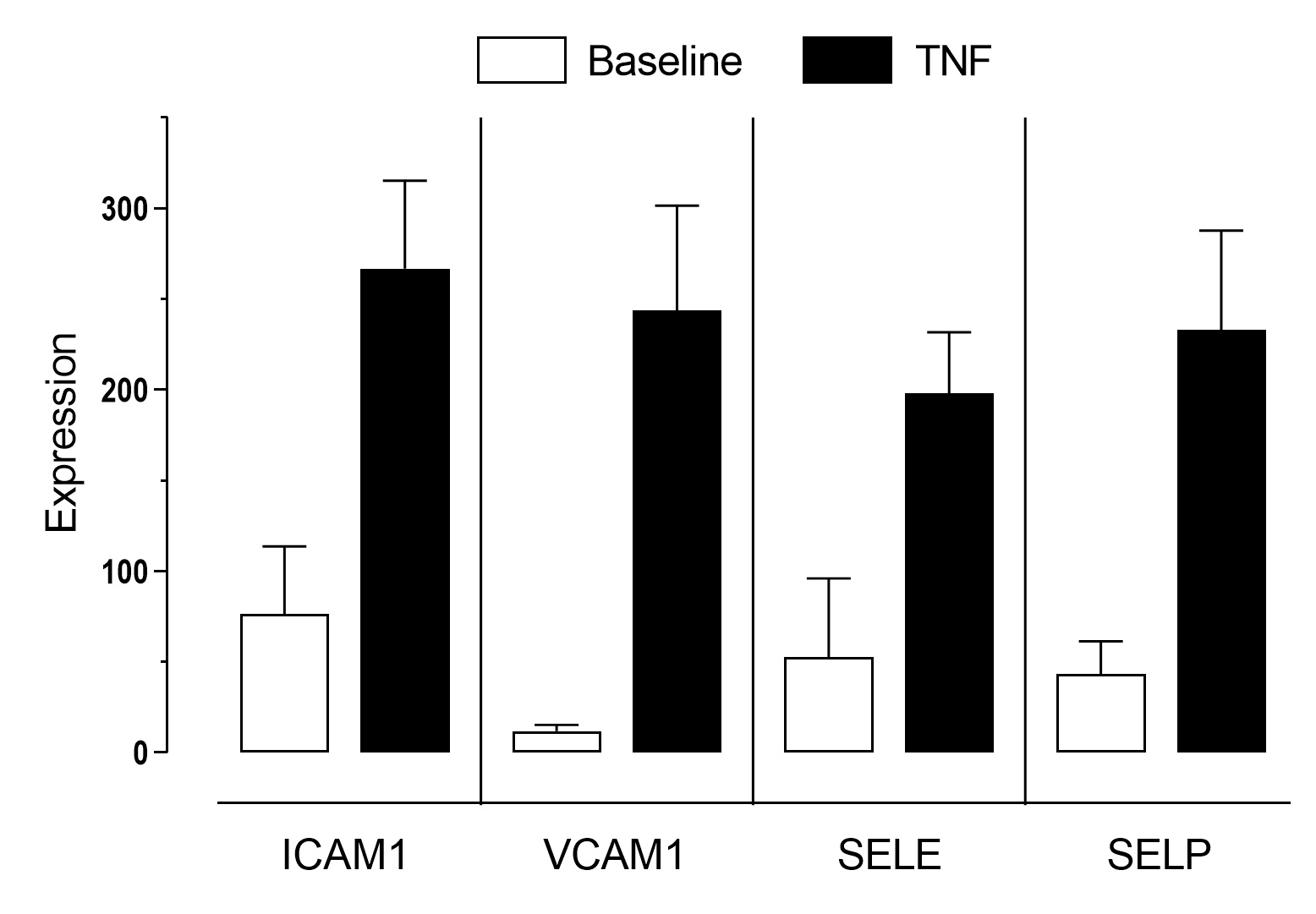

Supplement: Supplementary file 2 — Supplementary file2 (JPG 105 KB) [file 380_2021_1908_MOESM2_ESM.jpg]
